# Selected biomarkers of oxidative stress in healthy Beagle dogs: A preliminary study

**DOI:** 10.3389/fvets.2023.1063216

**Published:** 2023-03-24

**Authors:** Mathilde Porato, Stéphanie Noël, Joël Pincemail, Adelin Albert, Jean-Paul Cheramy-Bien, Caroline Le Goff, Annick Hamaide

**Affiliations:** ^1^Clinical Department of Companion Animals, School of Veterinary Medicine, University of Liège, Liège, Belgium; ^2^Departments of Cardiovascular Surgery and Clinical Chemistry, University Hospital, Boulevard de l'Hôpital, Liège, Belgium; ^3^Biostatistics, University Hospital, Boulevard de l'Hôpital, Liège, Belgium; ^4^Department of Clinical Chemistry, University Hospital, Boulevard de l'Hôpital, Liège, Belgium

**Keywords:** dog, oxidative stress, isoprostanes, biomarkers, Beagle

## Abstract

**Introduction:**

While oxidative stress has been studied in pathologic conditions in dogs, data in presumably healthy dogs and standardized protocols are lacking. This work purposed to bridge the gap by presenting provisional physiological ranges for oxidative stress biomarkers in a group of Beagle dogs.

**Methods:**

Based on our long-standing clinical expertise in the field of oxidative stress, nine plasma biomarkers of oxidative stress were evaluated for their concentrations (mean ± SD) in 14 healthy adult Beagle dogs.

**Results:**

Selected biomarkers were: vitamins C (7.90 ± 1.36 μg/mL) and E (34.1 ± 6.63 μg/mL), zinc (0.80 ± 0.17 mg/L), copper (0.54 ± 0.048 mg/L), selenium (256 ± 25.7 μg/L), total and oxidized glutathione (822 ± 108 μM and 3.56 ± 1.76 μM), myeloperoxidase (67.4 ± 56.2 ng/mL), and isoprostanes (340 ± 95.3 ng/mL). Glutathione peroxidase activity and superoxide anion production in whole blood were also measured. Glutathione peroxidase activity was 473 ± 34.0 IU/g of hemoglobin and superoxide anion production in whole blood was 18,930 ± 12,742 counts per 30 min. Reduced glutathione/oxidized glutathione and copper/zinc ratios were, respectively, 280 ± 139 and 0.70 ± 0.15. Sex-related differences were recorded for zinc (*p* = 0.0081), copper/zinc ratio (*p* = 0.0036) and plasma isoprostanes (*p* = 0.0045).

**Conclusion:**

Provisional physiological norms covering 95% of our group were proposed for each biomarker and should be of interest for future studies of canine oxidative stress.

## 1. Introduction

Oxidative stress has been defined as an imbalance between oxidants (e.g., free radical species derived from oxygen) and antioxidants in favor of oxidants, leading to a disruption of redox signaling and/or molecular damage to lipids, DNA and proteins (1). This definition takes into account both physiological and pathological effects of reactive oxygen species (ROS) (2). Produced in excess, ROS (including free radical (superoxide anion, hydroxyl radical) and non-free radical (hydrogen peroxide, hypochlorous acid) entities) induce irreversible oxidative damages to lipids, DNA and proteins that were associated with the development of diseases in humans (3). By contrast, ROS may also have important physiological actions when produced in low concentration, ROS may act as second messengers capable of regulating apoptosis, activating transcription factors and modulating the expression of various genes involved in the immune response (3). To regulate the excess of ROS production, both human and animal organisms respond with antioxidants (vitamins A, C and E, carotenoids, glutathione, ubiquinone and polyphenols), antioxidant enzymes (superoxide dismutase which catalyzes the dismutation of superoxide anions to hydrogen peroxide, glutathione peroxidase which catalyzes the reduction of several hydroperoxides to H_2_O *via* oxidation of reduced glutathione), trace elements (copper, zinc, and selenium), iron chelators and inhibitors of biological systems producing ROS (4).

Because ROS are short half-life molecules, the accurate detection of oxidative stress status (OSS) may reveal technically challenging. Moreover, it must be kept in mind that a single test does not reflect the presence of OS. Thus, in human studies, the first step to assess OS requires the use of a large battery of assays including determination of enzymes and low molecular weight antioxidants, analysis of trace elements, evidence of oxidative damages to lipids, proteins or DNA and identification of the sources responsible of high ROS production (5). The second step includes careful pre-analytical treatment of the sample since it may lead to artifactual values in the event of non-compliance with the protocol. This is of primordial importance to determine values for blood or urine OS biomarkers and, therefore, to correctly interpret the results (6). Such a rigorous methodology often lacks in canine research.

In dogs, several studies have evidenced increased OS in systemic diseases such as congestive heart failure, anemia, atopic dermatitis, mammary carcinoma, spinal cord injury or inflammatory bowel disease (7–16) when compared to healthy controls. More precisely, ill dogs have increased oxidant marker levels while endogenous antioxidants and biomarkers of the oxidant response concomitantly decrease in whole blood (12, 17–21), in serum (10, 13, 18, 20, 22), in plasma (12, 13, 17, 18, 20, 22), in urine (11) or in cerebrospinal fluid and spinal cord tissue (11). Other studies have described the values of several OS blood biomarkers in populations of healthy dogs from different breeds (17, 23) or from a single breed but at different ages (18), with the intent of addressing the effects of sex, age, and/or nutrition discrepancy. Despite these observations, basic information about physiological intervals of OS biomarkers in dogs is still lacking especially according to different breeds. Conversely, clinical studies rather defined a control population composed of healthy dogs of different environments, breeds, sex, reproductive status and ages that do not always match the study population. Consequently, and in spite of scientifically robust study designs, comparison between studies is limited whereas many of them tend to demonstrate that ill dogs experience an OS comparable to healthy subjects (8–11, 13, 24). Values obtained from homogeneous groups of Beagle dogs, with rigorous pre-analytical steps, would be beneficial to determine the baseline in a common breed, but to our knowledge, such data is lacking. This breed has already been chosen for the validation of OS biomarkers assays (erythrocyte osmotic fragility and detergent sensitivity to assess plasma alpha-tocopherol concentration) (19) and to determine the best storage conditions of serum for postponed measurement of OS biomarkers (20). The investigation of age-related and sex-related lipid peroxidation has also been made by comparing OS biomarkers in whole blood of different groups of Beagle dogs (22). The effect of vitamin C and E supplementation on blood OS biomarkers was also tested in this breed (21).

Reliable inter-individual physiological margins for OS biomarkers can be provided by thorough fundamental studies performed in a standardized population. Based on a highly-qualified expertise in humans, we initiated the present study to determine the OS biomarkers level in healthy Beagle dogs, respectively non-enzymatic anti-oxidants (glutathione, vitamins C and E), enzymatic antioxidant (glutathione peroxidase), trace elements (copper, zinc and selenium), inflammatory markers (myeloperoxidase and neutrophil activation) and specific markers of lipid peroxidation (isoprostanes). This allowed us to estimate mean and dispersion values and to propose provisional physiological norms for all selected OS biomarkers in our healthy dogs. Our results may serve as a reliable basis for future studies involving dogs, especially Beagle dogs.

## 2. Materials and methods

### 2.1. Animals

Six healthy intact female Beagle dogs and eight healthy intact male Beagle dogs were included in the study. Females belonged to 4 different litters and males belonged to two different litters. Dogs were 4.1 ± 0.8 years old. They were born and housed at the local animal facilities and were kept in the same kennel, had unrestricted access to water and were fed with commercial dry food. Male and female dogs had different diets to provide a diet with a lower lipid content to female dogs (Light weight care medium, Royal Canin^®^) compared to males (Adult Beagle, Royal Canin^®^). Dogs did not receive any oral anti-oxidant supplementation. Diet composition in relation to requirement for dogs is presented in Table 1. Females were in the anestrus phase of the estrus cycle at the time of sample collection. The animal study was reviewed and approved by Commission d'Ethique Animale de l'Université de Liège (reference 2123, May 2019).

**Table 1:** Diet composition and recommended nutrient levels for dogs according to the European pet food industry federation guidelines (2020), based on metabolizable energy requirement of 110 kcal/kg^0.75^.

**Nutrient**	**Adult maintenance minimum (per 100 g of DM)**	**Diet composition: male dogs (per 100 g of DM)**	**Diet composition: female dogs (per 100 g of DM)**
Protein (g)	18	27	27
Fat (g)	5.5	12	11
Eicosapentaenoic + Docohexaenoic acid	ND	0.3	0.24
**Minerals**			
Iodine (mg)	0.11	0.46	0.39
Iron (mg)	3.6	18.8	13.8
Manganese (mg)	0.58	6.9	5.9
Copper (mg)	0.72	1.5	1.5
Zinc (mg)	7.2	17.4	17
Selenium (μg)	18	38	35
**Vitamins**			
Vitamin A (IU)	606	3,050	2,350
Vitamin D (IU)	55.2	80	100
Vitamin E (mg)	3.6	58	43

For both diets, minerals and vitamins are reported as the additives. Nutrient requirements are indicated on a dry-matter basis and are per 100 g of diet.

ND, not determined; DM, dry matter.

### 2.2. Samples collection and analysis

Venous blood samples were collected from 8 a.m. to 9 a.m. from fasted animals in vacutainer tubes containing Na heparin or EDTA according to the tested parameter. All dogs were fasted at least 12 h before blood sampling. External jugular venipuncture was performed with a 21-gauge needle, and blood samples were collected in the appropriate tubes. Part of whole blood was removed for reduced and oxidized glutathione and glutathione peroxidase determination. Then both tubes were centrifuged at 1,500 g during 10 min. 500 μL of plasma EDTA were immediately added to 500 μL metaphosphoric acid (10%) for vitamin C stabilization and then frozen on dry ice. Another aliquot of 1 mL plasma EDTA was added to 5 μL butylhydroxytoluene at a concentration of 2 mg/mL in ethanol for the measurement of isoprostanes and then frozen on dry ice. The rest of EDTA plasma and serum were then discarded into separated tubes for analysis of vitamin E, myeloperoxidase, copper, zinc and selenium and frozen on dry ice. All samples were then stored at −80°C until analysis of oxidative stress biomarkers.

#### 2.2.1. Glutathione

Reduced (GSH) and oxidized (GSSG) levels were measured in whole blood using the BIOXYTECH^®^ GSH/GSSG-412™ assay kit (OxisResearch™, Oxis Health Product, Inc., Portland, OR, USA). Briefly, 50 μL of whole blood were collected and immediately stored at −80°C for total glutathione (GSHt) measurement. For oxidized glutathione (GSSG) measurement, 100 μL whole blood was added to 10 μL 1-methyl-2-vinylpyridinium trifluoromethanesulfonate to avoid glutathione oxidation and then immediately stored at −80°C. The method employs Ellman's reagent (5,5'-dithiobis-2-nitrobenzoic acid), which reacts with GSH to form a spectrophotometrically detectable product at 412 nm. GSSG can be determined by the reduction of GSSG into GSH. The following formula was used to calculate the reduced/oxidized glutathione ratio: *GSH/GSSG* = *((GSHt* −*2GSSG)/GSSG)*.

#### 2.2.2. Vitamin C

Plasma vitamin C was determined by a spectrophotometric method (520 nm) using the reduction of 2,6-dichlorophenolindophenol (Perkin Elmer Lambda 40, Norwalk, CT, USA) (25).

#### 2.2.3. Plasma isoprostanes

Plasma samples added to butylhydroxytoluene were purified through an original solid-liquid extraction protocol (26). After evaporation to dryness and reconstitution, samples were directly injected into a Waters XevoTM TQ-S mass spectrometer (Milford, MA, USA) equipped with an electrospray ionization source operating in positive ion mode and interfaced with a Waters Acquity UPLC I-Class inlet system (Miflord, MA, USA). Data acquisition was achieved using MassLynx Version 4.2 software (Milford, MA, USA) and TargetLynx version 4.2 software to Tandem Mass Spectrometry (UHPLC/MS-MS) method (Miflord, MA, USA).

#### 2.2.4. Vitamin E

Analysis of vitamin E (alpha and gamma-tocopherol) was performed by HPLC procedure (Alliance Waters, Washington, DC, USA) coupled with a diode array detector (PDA 2996, Waters, Milford, MA, USA) using Chromsytems kits (32,000, 34,000, and 68,000) (Chromsystems, Gräfelfing-Munich, Germany).

#### 2.2.5. Copper, zinc, and selenium

Copper, zinc, and selenium were detected in serum by inductively coupled plasma-mass spectroscopy (27).

#### 2.2.6. Enzymes

Glutathione peroxidase (GPx) activity was determined in 100 μL whole blood using Ransel kit (Randox Laboratories Ltd, County Antrim, New England). Briefly, GPx catalyzes the oxidation of GSH by cumene hydroperoxide. In presence of glutathione reductase and NADPH, the oxidized glutathione (GSSG) was converted to the reduced form with a concomitant oxidation of NADPH to NADP^+^. The decrease in absorbance at 340 nm was measured. Values expressed as International Unit (IU) were standardized to hemoglobin (IU/g Hb).

Plasma myeloperoxidase was assessed using the specific ELISA kit CSB-E12762C developed by Cusabio, Houston, TX, USA (detection range: 31.25–2,000 ng/mL; intra-assay precision: CV < 8%; inter-assay precision: CV < 10%).

#### 2.2.7. Neutrophils

Neutrophil count was measured with a commercial veterinary analyzer (Procyte Dx, IDEXX Laboratories, Inc, Westbrook, USA).

#### 2.2.8. Superoxide anion production in whole blood

Determination of superoxide anion production by NADPH oxidase in blood was assessed by the protocol described by Baptista et al. (28). Using 96 well plate, superoxide anion production was determined in 200 μL whole blood, collected in tubes containing EDTA as anticoagulant and diluted in 820 μL Dulbecco's Modified Eagle Medium and 200 μL lucigenin (1.5 x 10 μM). After 20 min of incubation at 37°C under gentle agitation, whole blood was stimulated by using phorbol myristate acetate (at the concentration of 0.1 μM) as a specific activator of NADPH oxidase, leading to superoxide anion production. The luminescence was immediately recorded at 37°C during 30 min using a luminometer GloMax^®^ Multi Microplate Multimode (Promega, Madison, WI, USA). Phorbol 12-myristate 13-acetate (PMA), N,N' Dimethyl-9,9'-biacridinium dinitrate (lucigenin) and fluorescein sodium salt were purchased from Sigma-Aldrich (St Louis, MI, USA).

### 2.3. Statistical analysis

Results were expressed as mean and standard deviation (SD). The Shapiro-Wilk (SW) test was used to assess the normality of the distribution of parameters. When significant, values were log-transformed to normalize their distribution. Mean values of male and female dogs were compared by the non-parametric Kruskal-Wallis (KW) test. Provisional physiological ranges containing 95% of the dogs were determined by computing 95%-content tolerance intervals at the 95% confidence level according to Guttman (29) and Eisenhart et al. (30). Specifically, given the mean m and the standard deviation SD of a sample of size N, the 95% confidence 95%-content tolerance interval is determined by computing the lower and upper limits m ± K.SD, where K values can be found in a published table (30). As an example, for *N* = 14, K = 3.012. Actually, as N tends to infinity, K gets closer and closer to the traditional 1.96 critical point of the standard Normal distribution. Results were considered significant at the 5% critical level (*P* < 0.05). Calculations were performed with R (version 4.1.0, Vienna, Austria).

## 3. Results

### 3.1. Dogs

The mean body weight of the dogs was 15.8 ± 1.2 kg in females and 17.3 ± 1.5 kg in males (*p* = 0.017). The body condition score (BCS, Body condition score chart, Ralston Purina Company, St Louis, MO) averaged 6.5 ± 1.2 in females and 5.3 ± 0.7 in males. All dogs, excepted females 9 and 11, had a BCS between 4 and 6. There was no significant difference in BCS between males and females.

The healthiness of the dogs was confirmed by the absence of illness history during the 2 weeks preceding the study, by a normal physical examination, normal complete blood count, normal serum biochemical analyses and normal urinalysis obtained during the week preceding the study. Specifically, plasma cholesterol concentration was similar to previously reported range (92–324 mg/dL) (31).

The diet was produced by a manufacturer according to the European Pet Food Industry Federation guidelines. These European guidelines are adapted from the guidelines of the National Research Council.

### 3.2. Oxidative stress biomarkers

The distribution characteristics of all oxidative stress parameters are given in Table 2. Three parameters did not follow a Gaussian distribution, namely vitamin E (*p* = 0.0059), myeloperoxidase (*p* = 0.0004) and to a lesser extent superoxide anion production in whole blood (*p* = 0.012). A log-transform was applied to normalize their distribution. Of note, for myeloperoxidase, given the presence of 8 right censored values (< 31 ng/mL), the tolerance interval was based on the median (rather than the mean) and the SD calculated from the Tukey formula SD = 0.74 IQR, where IQR is the interquartile range.

**Table 2:** Distribution characteristics and provisional 95%-content interindividual physiological intervals of oxidative stress parameters in presumably healthy Beagle dogs (*N* = 14)^*^.

**Parameter^#^(units)**		**Mean ±SD**	95%-Content interval^**^
Cholesterol (g/L)		1.86 ± 0.43	0.56–3.2
**Antioxidants**			
Vitamin C (μg/mL)		7.9 ± 1.36	3.8–12.0
Vitamin E (μg/mL)		34.1 ± 6.63	19.5–57.6
Vit E/Cholesterol (mg/g)		18.5 ± 1.96	12.6–24.4
**Glutathione**			
GSH_t_ (μM)		822 ± 108	496–1,147
GSSG (μM)		3.56 ± 1.76	0.0–9.0
GSH/GSSG		280 ± 139	0–710
GPX/Hb (IU/g)		473 ± 34.0	370–576
**Trace elements**			
Copper (mg/L)		0.54 ± 0.048	0.40–0.69
Zinc (mg/L)	Male Female	0.80 ± 0.17 0.70 ± 0.094 0.94 ± 0.14	0.30–1.3 0.35–1.1 0.31–1.6
Copper/Zinc ratio	Male Female	0.70 ± 0.15 0.80 ± 0.10 0.58 ± 0.11	0.24–1.2 0.43–1.2 0.09–1.1
Selenium (μg/L)		256 ± 25.7	178–333
**Markers of lipid peroxidation**			
Isoprostanes (ng/mL)	Male Female	340 ± 95.2 278 ± 70.1 424 ± 45.3	53.5–627 16.3–539 224–624
**Inflammation markers**			
Myeloperoxidase (ng/mL)		67.4 ± 56.2	2.9–335
Superoxide anion production in whole blood (CP30M)		18,930 ± 12,742	2,043–125,218

^*^N = 14 dogs (eight males and six females); four female dogs missing for Chemo luminescence and one female dog missing for Glut OX GSSG and GSH/GSSG. ^**^Physiological margins derived by Guttman 95% tolerance intervals at the 95% confidence level; margins are given for males and females in case of sex differences; margins for Vitamin E, Myeloperoxidase and Superoxide anion production in whole blood were derived from log-transformed values. ^#^All parameters are measured in plasma, excepted GPX/Hb (whole blood) and Superoxide anion production in whole blood. GSHt, total glutathione; GSH, reduced glutathione; GSSG, oxidized glutathione; GPX/Hb, glutathione peroxidase/hemoglobin; CP30M, count per 30 min.

Plasma concentrations in vitamins C and E, total glutathione, oxidized glutathione, copper, selenium, myeloperoxidase, the vitamin E/cholesterol ratio and glutathione peroxidase activity did not differ between males and females. Plasma concentrations in zinc were significantly higher in females than in males (*p* = 0.0081) and similarly for isoprostanes (*p* = 0.0045). By contrast, the plasma copper/zinc ratio was significantly higher in males compared to females (*p* = 0.0036). Superoxide anion production in PMA whole blood test did not differ between males and females.

Using mean and SD values, provisional physiological norms were determined for each OS biomarker by using the 95%-content tolerance intervals method. These norms are displayed in Table 2 not only globally but also according to sex when differences were observed between males and females.

## 4. Discussion

Levels of OS biomarkers in canine plasma or urine may vary with age (22, 32), sex (22, 23, 33), diet (18) or with the amount of time spent by the dog in the veterinary facility before sampling (34). Details about pre-analytical sample collection and handling are not always mentioned so that doubt regarding potential artifactual oxidation can raise. Moreover, methodology varies widely between studies, further preventing comparisons within literature data.

The definition of a reference population is of major concern when reporting biological data, in particular when it comes to compare study results. Most studies are clinical studies, dealing with a heterogeneous population of healthy dogs as a control/baseline group, with subjects of different breeds, age, sex and sexual status, which induces a great variability within results. For example, Freeman et al. reported OS biomarkers concentrations in a population of dogs from several breeds (4 to 71 kg) and of different ages (8.8 ± 2.6 years old) to compose the healthy group (7). In other studies, Beagle (19, 20, 22) and Labrador (18) dogs were favored. Mostly, the groups of healthy dogs that serve as control groups include about 10 to 20 dogs of different breeds, ages and sexes (9, 11, 33, 35). We chose a group of intact young adult Beagle dogs, which represent a typical group of interest in veterinary canine fundamental research, with individuals of medium size and good temper. We assessed OS biomarkers from this homogenous group of dogs with a validated protocol and standardized pre-analytical steps. Under these conditions, we demonstrated (i) that the methodology is deeply involved in the consistency of the results, which should encourage future studies to apply such a protocol, and (ii) that our results are highly valuable in the field of fundamental research on canine physiopathological mechanisms, where reliable physiological margins for OS biomarkers are absent.

The values of OS biomarkers were similar in male and female dogs, except for zinc and isoprostanes plasma concentrations and for copper/zinc ratio. The difference in plasma zinc concentration between males and females was not attributable to the difference in diet since zinc represented, respectively 17.4 mg and 17 mg per 100 g of dry matter in the diet received by both groups. The females in the present study were in the anestrus phase of the reproductive cycle to reduce the potential hormonal influence on plasma zinc concentration, as observed in women. Indeed, in women, the changes in plasma zinc concentration are positively correlated with the changes in plasma estradiol concentration during the menstrual cycle (36). Despite the sex-related difference and despite lower copper and zinc plasma concentrations in our dogs, copper/zinc ratio was similar to the ratio obtained in a heterogenous population of dogs of different breeds (37). Copper and zinc are mineral micronutrients acting as cofactors of certain endogenous antioxidant enzymes (37, 38). In human studies, the copper/zinc ratio is related to the systemic oxidative status: the higher the ratio, the more lipidic peroxidation due to high copper concentration (39).

Selenium intake was 3 μg per 100 g of dry matter lower in females than in males. It was therefore important to check if this could impact the levels of the other OS biomarkers. Levels in selenium (*p* = 0.33), but also those of glutathione peroxidase (*p* = 0.25) requiring this trace element for its antioxidant activity, were perfectly similar in both groups. It is therefore unlikely that the slight difference in selenium intake between the two groups will have an impact on the concentration of the other OS biomarkers. A similar conclusion can be drawn for iron. Indeed, about 70% of body's iron is found as hemoglobin in the red blood cells. There was no difference in hemoglobin levels between males (17.7 ± 1.3 g/dL) and females (16.8 ± 1.5 g/dL) (*p* = 0.55). Therefore, we concluded that even if the diet was different between males and females regarding selenium and iron composition, this should have no impact on the concentration of the other OS biomarkers.

Isoprostanes, as lipid peroxidation products, have been measured in plasma (7, 40) and in urine of dogs (8, 9, 11, 12, 15, 35) with conflicting results, due to different methods of analysis and ways to express the results. Indeed, immunoassays have been reported to lack consistency and specificity both in human and veterinary studies (41). For example, plasma median values 10,000 times lower than ours have been reported by Freeman et al. (median 0.02529 ng/mL, range 0.0111–0.0804 ng/mL) (7). Such a discrepancy is due to assay inaccuracy and to pre-analytic methodology. Indeed, in that study, plasma isoprostanes were measured with a commercial ELISA kit and samples were processed within 15 min. Conversely, our results rely on immediate processing and standardized analytic methods (UHPLC/MS-MS) as used in human studies (26, 28). Recently, Putman et al. concluded that the measurement of isoprostanes in veterinary medicine should preferably be done by UHPLC/MS-MS method rather than using ELISA kits (41). The reasons are twofold: (1) consistent overestimation of isoprostanes concentrations in ELISA test due to cross-reactivity with related compounds such as alternative isoprostanes isomers and prostaglandins; (2) poor agreement between different ELISA tests but also with LC/LC-MS/MS. Although the measurement of isoprostanes by ELISA tests is less expensive than that by UHPLC/MS-MS and less time consuming, the latter should be favored especially if high precision is desired (definition of diagnostic cut-off values for example) or if dilutions are required with ELISA kits. It is therefore important to encourage researchers to seek for this possibility whenever possible, because it may represent a specific interest in the future. The hypothesis of methodology discrepancy can also be formulated to explain the extreme variations found in the literature for isoprostanes urinary concentration, with values of 0.00714 ng/mL (8) and 399 pg/mL (9) (median and mean, respectively). Moreover, isoprostanes are detectable in all fluids and tissues of mammals but they may not match with the systemic isoprostanes concentration (41). In our study, assays were also performed but were unsuccessful in determining isoprostanes in urine samples due to the presence of impurities in the matrix interfering with the elution peak. In human studies, a strong positive correlation has been reported between copper/zinc ratio and lipid peroxidation products (42, 43). In our dogs, this was not the case for unexplained reason (isoprostanes and copper/zinc ratio: *r* = −0.57, *p* = 0.033, data not shown).

Vitamin C, or ascorbate, is a hydro-soluble ubiquitous carbohydrate and a powerful antioxidant acting as a scavenger of several free radicals and ROS. It can regenerate other molecules from their oxidized form, such as vitamin E, hence inhibiting lipid peroxidation as well (44). Moreover, vitamin C synthesis is possible in all species except in higher-order primates, in guinea pigs and in some bat, fish, and bird species (44). While analytic methods were different, plasma concentrations in vitamin C in the present study were similar compared to the study of Freeman et al. (median 7.2 μg/mL, range 4.3–8.5 μg/mL) in which dogs were fed with commercial food (7). Vitamin E, represented by eight molecules (four tocopherols and four tocotrienols) is a potent antioxidant, made by plants, with lipoperoxyl radical scavenging activities (45). We observed higher plasma concentrations in vitamin E compared to the study of Freeman et al. (median 0.0282 μg/mL, range 0.0174–0.0378 μg/mL) (7). This difference could be attributable to the dosage of alpha-tocopherol only by Freeman et al. It may also be due to differences in the pre-analytical steps, since dosage method was similar to ours. This is further highlighted by the variability between other studies in which control groups are, respectively composed of 14 and 10 adult dogs of different breeds and sexes: median 55 μg/mL, range 18–88 μg/mL by Viviano and VanderWielen (9) and median 24.1 μg/mL, range 16.8–31.2 μg/mL by Verk et al. (33). As a labile compound, dosage of vitamin E relies on sample process and delay during the pre-analytical steps may compromise the quantity of vitamin E in the sample. Moreover, food type and modalities of food preservation might have influenced vitamin E intake by the dogs, contrary to the dogs of our study who were fed the same food. However, the major reason explaining such a high variability in all these studies (7, 9, 33) is due to the fact that the authors do not take in account the cholesterol concentration as we did in the present work. Indeed, we found a highly positive correlation (*r* = 0.91, *p* < 0.0001) between both plasma vitamin E and cholesterol concentrations (data not shown), which was expected as it had already been reported in human (46).

Among OS biomarkers, glutathione is a thiol tripeptide synthesized in all mammalian cells. The blood concentrations in oxidized glutathione (median 0.08 μM, range 0.01–0.29 μM) reported by Freeman et al. (7) were lower than ours which is surprising. Indeed, the population of dogs in the study of Freeman et al. was older (8.8 ± 2.2 years old) compared to our subjects. Consequently, higher blood concentrations in oxidized glutathione would have been expected, as it has been described in elderly (47). Again, this illustrates the divergences of the results when obtained from studies built on different protocols and conducted on different populations. The cellular balance of the redox couple formed by glutathione and its oxidized disulfide form provides a dynamic indicator of OS. During acute OS, reduced glutathione concentration decreases and the concentration of its disulfide form increases (24, 48). Glutathione peroxidase is a selenium-dependent enzyme responsible for glutathione oxidation coupled with organic peroxides reduction. Its value in our study was similar to values previously reported in a population of 30 healthy male and female dogs aged 7 months to 11 years old (454 (349–705) IU/g Hb) (17) and was not affected by the difference in diet composition between males and females. Similarly, in the study of Tomsič et al. glutathione peroxidase activity did not differ between males and females despite the different diet provided by the owners (17). A comparable range (394 ± 44 IU/g Hb) was also found in 10 healthy adult dogs (4.4 ± 2.5 years of age) of different breeds and sexes (33).

Myeloperoxidase is an inflammatory marker which illustrates the capacity of a system in producing ROS, such as hypochlorous acid. It is produced in both neutrophils and monocytes. Our plasma levels in myeloperoxidase were higher than those previously reported (0.2733 ± 0.1796 ng/mL) in a population of healthy small-breed dogs, aged from 7 to 14 years old (49). In the same study, myeloperoxidase levels were higher in healthy dogs than in dogs with a cardiac disease, which contrasts with the trends observed in humans with cardiovascular diseases (50).

To our knowledge, canine neutrophil activation has never been reported in a standardized group of dogs and with the protocol we used. In the study of Almeida et al. neutrophil activation was determined through superoxide production using the nitroblue tetrazolium reduction test in isolated neutrophils in the presence or absence of phorbol 12-myristate 13-acetate (PMA) (51). Silva et al. estimated neutrophil activation by measurement of the fluorescence in a capillary flow cytometer of isolated neutrophils in the presence or absence of PMA (52). These different protocols do not allow comparison of neutrophil activation between studies.

The small number of dogs (*N* = 14) included in this study is a limitation, in particular when it comes to establish reference norms according to the recommendations of the American Society of Veterinary Clinical Pathology for de novo reference interval definition (53). The method of 95%-content tolerance intervals at specific confidence levels provide an interesting alternative to conventional reference intervals; they have been widely used in industry and can be determined regardless of the sample size. Thus, they can operate as provisionary working reference norms and may be regularly updated as more animals become available. When a population of < 40 individuals is studied, the American Society of Veterinary Clinical Pathology advises to emphasize on the subjects and to adhere to standardized collection techniques and well-controlled methods of analysis (53). This study purposed to determine the OS status in healthy Beagle dogs and to establish working interindividual physiological margins of OS biomarkers. Consequently, we selected a standard and homogeneous group of dogs to minimize the variation between animals and adhered to a methodology identical to human studies. Our results allow comparison with a similar population of healthy adult Beagle dogs but they preclude extrapolation to growing dogs or older dogs, as well as dogs with specific condition such as pregnant bitches, athletic dogs or specific breeds (such as brachycephalic dogs, Greyhounds).

## 5. Conclusion

The 95%-content physiological intervals we proposed for plasma concentrations in vitamin C, vitamin E, zinc, copper, copper/zinc ratio, glutathione, myeloperoxidase and isoprostanes have been defined in a standardized group of healthy Beagle dogs using highly specialized techniques of human medicine research. They represent provisional inter-individual physiological margins for future studies involving OS in dogs. Sex-related differences observed for zinc, copper/zinc ratio, and plasma isoprostanes should be interpreted with caution.

## Data availability statement

The original contributions presented in the study are included in the article/supplementary material, further inquiries can be directed to the corresponding author.

## Ethics statement

The animal study was reviewed and approved by Commission d'Ethique Animale de l'Université de Liège.

## Author contributions

Conceptualization: AH, JP, SN, and MP. Investigation: MP, JP, J-PC-B, SN, and CL. Writing—original draft preparation: MP. Writing—review and editing: JP, AA, SN, and AH. Funding acquisition: AH and SN. All the authors have read and agreed to the published version of the manuscript.
